# Genetic dissection of apoptosis and cell cycle control in response of colorectal cancer treated with preoperative radiochemotherapy

**DOI:** 10.1186/1471-2407-6-124

**Published:** 2006-05-10

**Authors:** Isrid Sturm, Beate Rau, Peter M Schlag, Peter Wust, Bert Hildebrandt, Hanno Riess, Steffen Hauptmann, Bernd Dörken, Peter T Daniel

**Affiliations:** 1 University Medical Center Charité, Campus Virchow Klinikum, Department of Hematology and Oncology, 13353 Berlin, Germany; 2 University Medical Center Charité, Campus Buch, Department of Surgery and Surgical Oncology, 13125 Berlin, Germany; 3 University Medical Center Charité, Campus Virchow Klinikum, Department of Radiology, 13353 Berlin, Germany; 4 University of Halle-Wittenberg, Institute of Pathology, 06097 Halle, Germany

## Abstract

**Background:**

In previous analyses we identified therapy-induced upregulation of the CDK inhibitor p21^CIP/WAF-1 ^and consequently decreased tumor cell proliferation or loss of Bax as adverse factors for survival in rectal cancer treated with radiochemotherapy. Here, we address the individual role of p53 and its transcriptional targets, p21^CIP/WAF-1 ^and Bax, on apoptosis induced by individual components of multimodal anticancer therapy, i.e. 5-fluorouracil (5-FU), ionising γ-radiation (IR) and heat shock/hyperthermia.

**Methods:**

We analysed tumor samples 66 patients with rectal carcinoma treated by a neoadjuvant approach with radiochemotherapy ± heat shock/hyperthermia for the expression and mutation of p53 and the expression of p21^CIP/WAF-1 ^and Bax. These data were correlated with the tumor response. The functional relevance of p53, p21^CIP/WAF-1 ^and Bax was investigated in isogeneic HCT116 cell mutants treated with 5-FU, IR and heat shock.

**Results:**

Rectal carcinoma patients who received an optimal heat shock treatment showed a response that correlated well with Bax expression (p = 0.018). Local tumor response in the whole cohort was linked to expression of p21^CIP/WAF-1 ^(p < 0.05), but not p53 expression or mutation. This dichotomy of p53 pathway components regulating response to therapy was confirmed in vitro. In isogeneic HCT116 cell mutants, loss of Bax but not p53 or p21^CIP/WAF-1 ^resulted in resistance against heat shock. In contrast, loss of p21^CIP/WAF-1 ^or, to a lesser extent, p53 sensitized predominantly for 5-FU and IR.

**Conclusion:**

These data establish a different impact of p53 pathway components on treatment responses. While chemotherapy and IR depend primarily on cell cycle control and p21, heat shock depends primarily on Bax. In contrast, p53 status poorly correlates with response. These analyses therefore provide a rational approach for dissecting the mode of action of single treatment modalities that may be employed to circumvent clinically relevant resistance mechanisms in rectal cancer.

## Background

The development of cancer is a multifactorial process that depends on alterations in both proliferation control and the cell death machinery. The disruption of these regulatory networks, especially of those involved in cell death signaling, plays a key role in the acquired or constitutive resistance of malignant tumors to cytotoxic anticancer therapies [1]. Disruption of components of the p53 signaling pathway is associated with a poor prognosis in gastrointestinal tumors [2-6]. Furthermore, in various cell line models, restoration of defective cell death signaling sensitized tumor cells to chemotherapy and irradiation [7-9].

Despite this progress in understanding the molecular basis of cancer and resistance to anticancer therapy there is so far only scarce evidence regarding the use of such molecular parameters in predicting the response to anticancer therapy. Therefore, the aim of this study was to dissect the role of transcription targets of p53, p21^CIP/WAF-1 ^(cell cycle regulation) [10] and Bax (pro-apoptotic multidomain Bcl-2 family member) [11], in cell death induced by the individual components of the multimodal neoadjuvant therapy applied in treatment of rectal cancer, i.e. 5-fluoruracil (5-FU), ionising γ-radiation (IR), and heat shock. These functional analyses were performed in isogeneic HCT116 cell mutants. In addition, samples from rectal cancer patients were investigated that were obtained prior to and after treatment with neoadjuvant radiochemotherapy and surgery to define the in vivo and clinical role of p53, p21^CIP/WAF-1 ^and Bax on the local tumor response.

Data presented here delineate a dichotomy regarding the impact of these p53 pathway components on cell death induction by 5-FU, IR and heat shock with p53/p21 regulating response to radiochemotherapy and Bax being especially critical in response to heat-shock.

## Methods

### Cell culture

HCT116 colorectal cancer cell line and HCT116 cells carrying a targeted knock out for the genes p53 (HCT116 p53^-/-^) [12], p21^CIP/WAF-1 ^(HCT116 p21^-/-^) [13] and Bax (HCT116 Bax ^-/-^) [14] were grown in RPMI (Invitrogen/Gibco, Karlsruhe, Germany) supplemented with 10% FCS, 100 U/ml penicillin and 0.1 μg/ml streptomycin. The genotype was routinely verified by standard immunoblotting procedures, as described [15].

### Measurement of cell death by flow cytometry

Apoptosis was determined on the single cell level by measuring the DNA content of individual cells on a FACScan (Becton Dickinson; Heidelberg, Germany). Briefly, cells were seeded at a density of 5 × 10^5 ^cells in 25 cm^2 ^plastic flasks. After 24 h, cells were exposed to 5-FU (Sigma, Germany) or IR from a ^137^Cs source. Heat shock was applied for 2 hours in 1°C steps. For the application of heat shock, the culture flasks were put in an incubator with humidified air and 5% CO_2 _for two hours. The temperature in the incubator was preset and unchanged during the procedure and was controlled by a digital thermometer before and during the procedure. Cells were harvested after 72 h. For measurement of apoptosis, the cellular DNA content was determined following fixation of the cells, digestion of RNA and staining of the genomic DNA with propidium iodide (PI) as described [16]. Cells were measured with logarithmic amplification in the FL-3 channel of a FACScan (Becton Dickinson; Heidelberg, Germany) equipped with the CELLQuest software. Data are given in % hypoploidy (sub-G1), which reflects the percentage of cells displaying apoptotic DNA-fragmentation.

### Patients

In this study, 66 patients with rectal cancer, endosonographically classified as uT3 (n = 58) or uT4 (n = 8) without distant metastases, underwent preoperative radiochemotherapy alone (RCT n = 35) or combined with regional hyperthermia (HRCT n = 31). Preoperative radiochemotherapy (RCT) was given with a daily fraction of 1.8 Gy on five days per week for five weeks (total dosis 45Gy) combined with 5-FU (300 mg/m2) plus leucovorin (50 mg) as a short infusion on the 1st until the 5th and 22nd until the 26th day. Hyperthermia was administered once a week utilising the annular-phased-array system BSD-2000 (BSD Medical Corp, Salt Lake City, Utah, USA) with the SIGMA-60 applicator. Immediately after completion of the hyperthermia session, the patient was exposed to the daily dose of irradiation (1.8 Gy). Curative surgery was attempted 4–6 weeks after completion of preoperative therapy. Patients characteristics and exact treatment modalities were as published previously [6]. Curative surgery was attempted 4–6 weeks after completion of preoperative HRCT, with sphincter sparing surgery in 42 cases (64%) and abdomino-perineal resection with total mesorectal excision in 24 cases (36%). In patients with suspected tumor infiltration in surrounding structures, these were included by applying an extended monobloc resection. The detailed study protocol is described in reference 17. In case of R0-resection, the patients received an additional 4 courses of 5-FU/Leucovorin as adjuvant therapy. According to the protocol, postoperative treatment was individualized for the 6 patients with R1 or R2 resection. Patients were followed for a median of 39.3 months (range 11.3–83.4 months). Follow-up was done in our outpatient clinic every 3 months for the first 2 years and every 6 months thereafter. **The institutional board of the medical faculty Charité Campus Berlin Buch approved this study. All patients gave their informed consent for tissue asservation and molecular analyses.**

### Tissue samples and pathology evaluation

Biopsies of the representative central tumor area of the rectal cancer were taken (by two investigators (B.R. and U.S.)) 1 week before and 4–6 weeks after therapy. After therapy, the tumor biopsy was taken from the specimen together with the pathologist. The percentage of tumor regression was determined by the ratio of necrotic or fibrotic areas to the area of intact cells upon histopathological examination of the resected material post radiochemotherapy. Furthermore, lymphatic and venous vessel infiltration by tumor cells was evaluated in the resected specimen after radiochemotherapy. R0-resection was defined as a tumor free distance to the circumferential resection margin above 1 mm and was microscopically assessed by the pathologist.

### Definition of response

Response to preoperative treatment was classified according to the WHO criteria [17] as described [6] utilising all imaging modalities (CT, MRI, endosonography) and endoscopy. Complete remission (CR) was defined after successful surgical resection by histopathological examination of the resected specimen for the absence of vital tumor cells. Partial remission (PR) was defined by a decrease in the depth of tumor infiltration, as determined by pathological examination, compared to the pretherapeutic uT-values (i.e. a reduction of T-category) or in cases in which the maximum tumor diameter (transverse or longitudinal) measured by endoscopy or CT/MRI showed a decrease of at least 50%. Responders in this study were those patients with a CR or PR, and nonresponders are all others. According to the defined criteria, 39 patients were responders and 27 patients were non-responders. The number of hyperthermia sessions per patient was 4.1 + 1.8. We found a significant relationship for the quality of hyperthermia, as determined by the parameter *cum min *T_90 _> 40.5°C (> 120 minutes for optimal hyperthermia, < 120 min for suboptimal hyperthermia), and treatment response (Table 1).

### Immunohistochemistry for Bax, p53 and p21^CIP/WAF-1^

Tumor specimen pre- and posttherapy were investigated by immunohistochemistry. Specimens of the representative central tumor area of the rectal cancer were taken 1 week before therapy and 4–6 weeks after neoadjuvant therapy. For immunohistochemistry, 4 μm slices from paraffin-embedded tissue were stained applying standard techniques [2]. The primary antibody was a mouse monoclonal antibody for Bax (clone YTH-2D2, Trevigen, Gaithersburg, MD, USA; dilution 1:750), a mouse monoclonal antibody for p53 (clone DO-7, Dako, Glostrup, Denmark; dilution 1:75), and a mouse monoclonal antibody for p21^CIP/WAF-1 ^(clone 6B6, Pharmingen, San Diego, CA, USA; dilution 1:75). Analysis of slides was done blinded, by two observers without knowledge of clinicopathological data. Four high power fields (400x) were evaluated for percentage positive cells (0–100 % in 5 % steps), and staining intensity (0 to 3). For further analysis, we used the staining index (STI = product of percentage positive cells (0–100%) and staining intensity (0, 1, 2 or 3)) as described [4,6]. The "dynamic expression" is calculated by the post-therapeutic minus the pre-therapeutic staining index, i.e. a value > 0 denotes an increase in expression post-therapy, whereas a value < 0 denotes a decrease (see Fig. 5).

### Statistical analysis

For intervariable assessment, the nonparametric U-test or the χ^2^-test were used. For longitudinal expression studies the difference of STI post-therapy and pre-therapy was calculated for paired samples. An increase in expression post-therapy is indicated by a higher "dynamic" STI compared to a decrease post-therapy, where the "dynamic" STI is negative. All tests were 2-sided and p < 0.05 was considered statistically significant. The analyses were done by the use of SPSS 10.0 and GraphPad Prism4 for Apple MacIntosh.

## Results

### Impact of Bax, p53 and p21^CIP/WAF-1 ^knock out in the HCT116 model

An increase in p21^CIP/WAF-1 ^expression in rectal carcinoma treated by preoperative multimodal treatment results in a significantly reduced disease free survival after multimodal therapy for rectal cancer [6]. This finding suggested a significant role for p21^CIP/WAF-1 ^in the development of resistance to therapy in rectal carcinoma. This resistance mechanism appeared to occur independently from the tumor suppressor gene p53 and its transcriptional target Bax. A similar effect connecting p21^CIP/WAF-1 ^and resistance to chemotherapy was previously demonstrated in the HCT116 cell line model where the targeted knock-out of the p21^CIP/WAF-1 ^gene results in sensitivity to DNA damaging chemotherapy [13]. To clarify the roles p53 and the p53 targets p21 and Bax, we exposed the colorectal cancer cell line HCT116 to the individual therapeutic modalities employed in clinical rectal carcinoma therapy: IR, 5-FU and heat shock. The benefit of this cellular system is that isogeneic sublines can be tested that carry a gene specific knock-out for either Bax, p53 of p21^CIP/WAF-1 ^[12-14].

**Figure 1:**
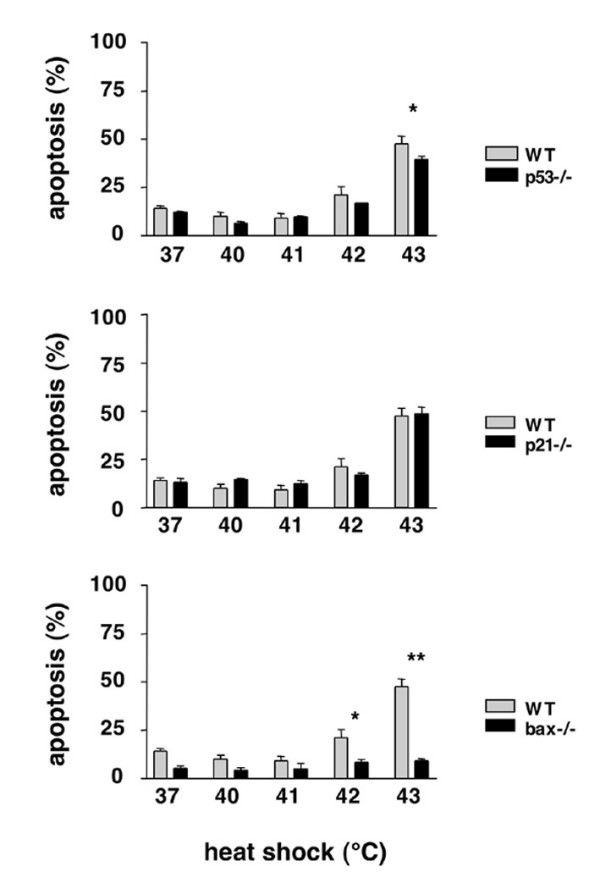
**Induction of apoptosis by heat shock. HCT116 cells were exposed to heat shock and cultured for 72 h. **Grey bars: wild type. Black bars: knock-out mutants. Upper panel p53^-/-^, middle panel p21^CIP/WAF-1-/-^, lower panel Bax ^-/-^. *: p < 0.05, **: p < 0.005.

**Figure 2:**
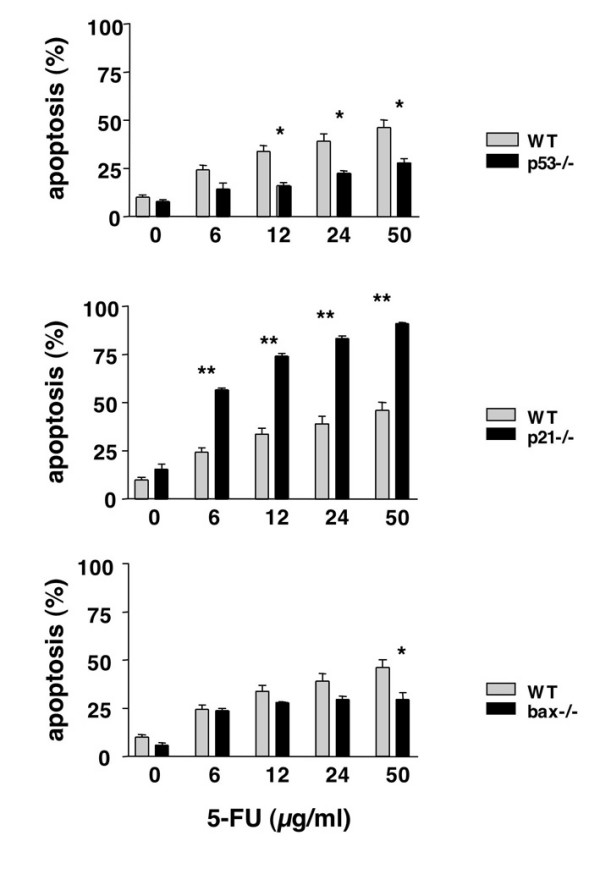
**Induction of apoptosis by 5-flurouracil. HCT116 cells were cultured for 72 h in the presence of 5-FU. **Grey bars: wild type. Black bars: knock-out mutants. Upper panel p53^-/-^, middle panel p21^CIP/WAF-1-/-^, lower panel Bax ^-/-^. *: p < 0.05, **: p < 0.005.

**Figure 3:**
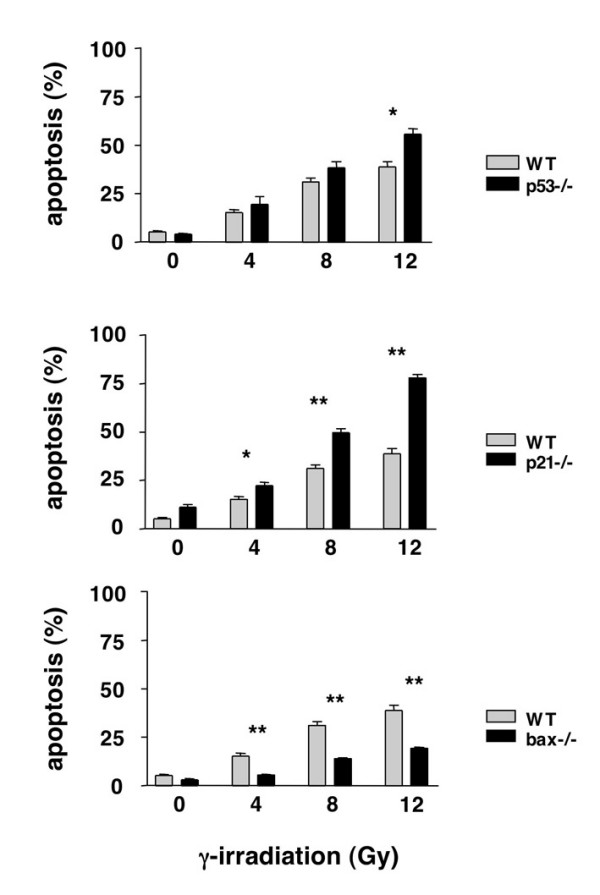
**Induction of apoptosis by γ-radiation. HCT116 cells were exposed to IR and cultured for 72 h. **Grey bars: wild type. Black bars: knock-out mutants. Upper panel p53^-/-^, middle panel p21^CIP/WAF-1-/-^, lower panel Bax ^-/-^. *: p < 0.05, **: p < 0.005.

All treatment modalities resulted in the dose dependent induction of apoptotic cell death after 72 h in the HCT116 wild-type cells. Notably, we observed differential effects of individual gene knock out on the cell death induction by the three modalities. Heat shock-induced apoptosis was entirely Bax-dependent: Bax deficient HCT116 cells were refractory to apoptosis induced upon treatment with heat shock at 42°C and 43°C for 2 h (Fig. 1). Thus, disruption of Bax renders HCT116 cells refractory for induction of apoptotic cell death following moderate heat shock. In contrast, 5-FU (Fig. 2) and IR-induced apoptosis (Fig. 3) were only partially impaired upon absence of Bax.

Loss of p53 resulted in a reduction of apoptosis (Fig. 2) following exposure to 5-FU that was, however, less pronounced as compared to loss of Bax or disruption of p21^CIP/WAF-1^. Notably, IR was not impaired upon loss of p53. There, loss of p53 led to a moderate increase of apoptosis induced by irradiation, presumably through the consequent loss of cell cycle checkpoint control [12]. This increase was, again, less pronounced as that observed in the p21^-/- ^HCT116 cells. Finally, loss of p53 resulted in a moderate reduction of heat shock induced apoptosis (Fig. 1).

In contrast to Bax and p53, loss of p21^CIP/WAF-1 ^led to an impressive sensitisation to 5-FU and irradiation-induced cell death (Fig. 2 and 3). Compared to 5-FU and IR, heat shock induced apoptosis was, however, not impaired upon loss of p21^CIP/WAF-1 ^(Fig. 1). This indicates that loss of cell cycle checkpoint control is important in sensitisation for irradiation and 5-FU but not for heat shock-induced apoptosis.

### Deregulation of Bax, p53, and p21^CIP/WAF-1 ^and clinical response

**Figure 4:**
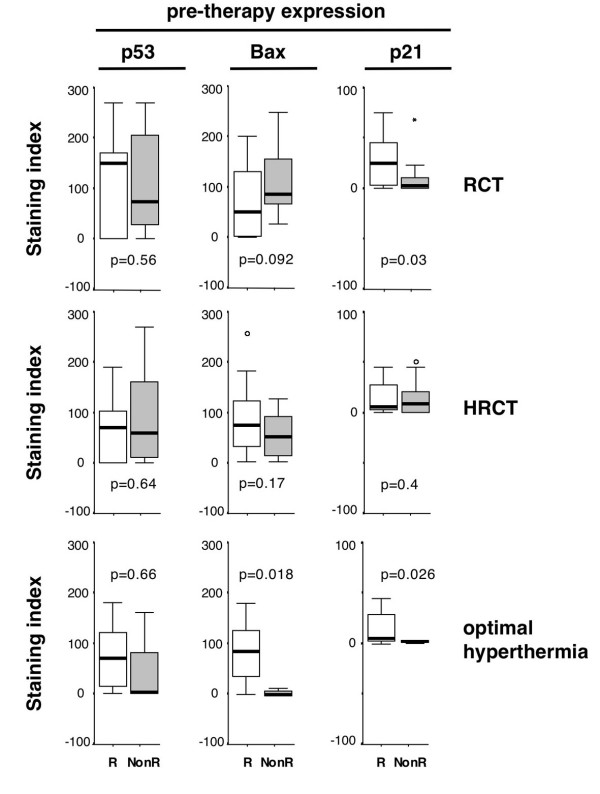
**Pre-therapeutic expression of p53, Bax, p21^CIP/WAF-1 ^and response to therapy in rectal carcinomas**. Box plot analysis of staining indices (STI) for p53, Bax and p21^CIP/WAF-1^. White bars: Responders (**R**), grey bars: Non-responders (**NonR**), RCT: radiochemotherapy. (responders n = 21, non-responders n = 14), HRCT: hyperthermic radiochemotherapy (responders n = 18, non-responders n = 13), optimal hyperthermia: subgroup of HRCT, definition see text (responders n = 13, non-responders n = 3).

To corroborate these functional data on the impact of distinct genotypes on response to cancer therapy, we analysed a cohort of 66 patients with a defined p53/Bax/p21 status who received 5-FU containing radiochemotherapy (RCT) alone or in combination with local heat shock (hyperthermia). Expression profiles were determined in both specimens obtained at the time of diagnosis (pre-therapy) and resection material obtained at the time of surgery, i.e. after pre-operative (neoadjuvant) radiochemotherapy (post-therapy). There, patients responding to RCT in combination with optimal hyperthermia had a significantly higher Bax expression level than non-responders (Bax staining index (STI) in responders: 73 (median, range 0–180) as compared to 0 (median, range 0–13) in non-responders (p < 0.02) (Fig. 4). Pre-therapeutic p21^CIP/WAF-1 ^expression level was correlated with the response in both the RCT group and the RCT-optimal hyperthermia group: p21^CIP/WAF-1 ^STI in responders: 25 (median, range 0–150) compared to 2.5 (median, range 0–70) in non-responders (p = 0.03) (RCT group) and p21^CIP/WAF-1 ^STI in responders: 6 (median, range 0–150) compared to 2 (median, range 0–3) in non-responders (p = 0.026) (RCT and optimal hyperthermia group) (Fig. 4). In contrast and in analogy to the HCT116 data, no correlation of the p53 expression levels to response state was seen. The same was true for p53 gene mutations that had no impact on response to therapy. We identified 8 patients with p53 gene mutations, as described [6]. Of those, 6 were found in tumors classified as „responder„, only 2 in tumors classified as „non-responder„ (data not shown). Thus, there was a trend for better tumor response to treatment in case of p53 mutation that failed, however, to reach statistical significance (χ^2^-test p = 0.56).

Thus, like in the situation of the HCT116 model, loss of Bax results in resistance to heat shock. Surprisingly, a response to RCT or HRCT was associated with higher p21 expression. In contrast, p53 deficiency had no impact on the clinical response to RCT or heat shock plus RCT.

**Figure 5:**
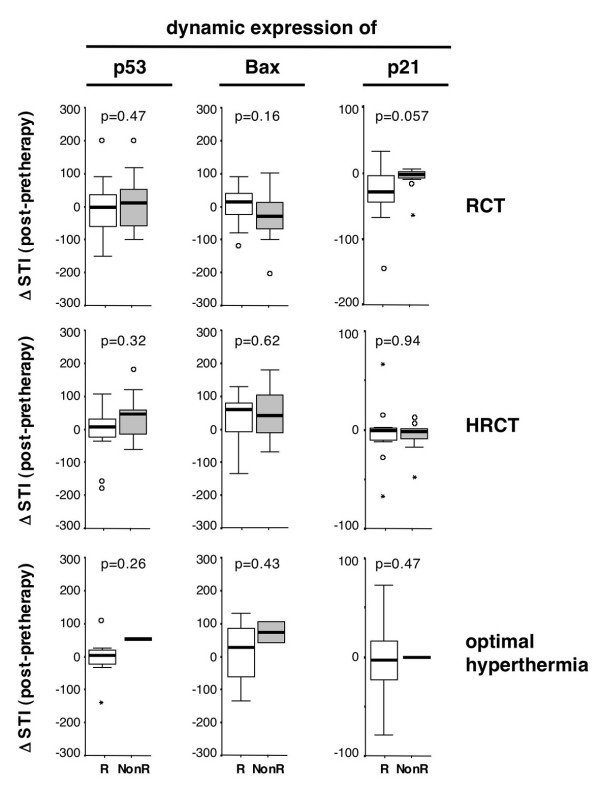
**Dynamic expression of p53, Bax, p21^CIP/WAF-1 ^and response to therapy in rectal carcinomas**. Box plot analysis of staining indices for p53, Bax and p21^CIP/WAF-1 ^(post-therapy staining index (STI) – pre-therapy STI. White bars: Responders (**R**), grey bars: Non-responders (**NonR**). RCT: responders n = 14, non-responders = 12, HRCT: responders n = 11, non-responders = 9, Optimal hyperthermia: responders n = 8, non-responders = 3.

To gain additional information on the kinetic behaviour of p53 signaling components, we examined tumor samples again at the time of surgery, i.e. 4–6 weeks after the completion of neoadjuvant therapy. Due to complete remission in some patients, the amount of paired samples (pre-and post-therapy) was reduced to 46 pairs. The post-therapy expression levels of Bax, p53 or p21^CIP/WAF-1 ^alone were, however, not significantly different between responder and non-responder groups. Upon comparison of pre-versus post-therapeutic expression levels, the subset of tumors with an increase of p21^CIP/WAF-1 ^expression showed a significantly reduced disease free survival [6]. We therefore investigated the impact of such dynamic changes in expression levels for Bax, p53 or p21^CIP/WAF-1 ^on the tumor response (Fig. 5). Besides a non-significant trend for higher p53 and Bax levels in the optimal hyperthermia/heat shock group, we found the dynamic increase of p21^CIP/WAF-1 ^expression in the RCT-treated group to be associated with a failure to respond to therapy. The median dynamic p21 STI for responders was lower (-28.25 (range -148.4 – 32.5; n = 14) than for non-responders (-1.75 (range -69 – 5.5); n = 12; p = 0.057) (Fig. 5). Hence, tumors of patients who responded to RCT exhibited a strong decrease of p21^CIP/WAF-1 ^expression post-therapy. This finding is in line with both the data from the HCT116 model and the observation that the dynamic increase of p21^CIP/WAF-1 ^as observed in paired tumor samples is related to a highly reduced disease free survival, i.e. a failure to respond to clinical cancer therapy [6].

## Discussion

Aim of this study was to investigate the p53 and to define the impact of individual gene defects in p53 and the transcriptional p53 targets Bax and p21^CIP/WAF-1^. To this end, we investigated HCT116 mutants derived from targeted gene knock-out of p53, p21^CIP/WAF-1 ^or Bax and validated the relevance of these data by the use of clinical samples of rectal carcinoma.

In the present study we show the relevance of the cyclin dependent kinase inhibitor p21^CIP/WAF-1 ^for apoptosis sensitivity towards 5-Fuand IR *in vitro *and for local tumor control *in vivo*. In HCT116 wild type and HCT116 p21^-/- ^knock-out cells, we found a strong correlation between disruption of p21^CIP/WAF-1 ^and *in vitro *sensitivity towards 5-FU and, to a lesser extent, towards IR-induced cell death. The lack of p21^CIP/WAF-1 ^in tumor cells was described to lead to an uncoupling of S- and M-phase and to cell death in consequence of a mitotic catastrophe upon DNA damage [13]. Conversely, sensitivity to IR in tumor cells is higher when the expression of p21^CIP/WAF-1 ^is blocked [18]. Our *in vitro *data are in accordance with these findings. A striking finding is the strong dependency of 5-FU induced cell death on p21^CIP/WAF-1^. To our knowledge, this is the first description of this effect. Furthermore, the *in vitro *experiments show that heat-shock induced apoptosis does not rely on p21^CIP/WAF-1^. This is a strong cell biological argument for the combination of different treatment modalities, i.e. the addition of hyperthermia (p21^CIP/WAF-1^-independent) to radiochemotherapy (p21^CIP/WAF-1^-dependent). In fact, these cell biological data are in accordance with the impact of p21^CIP/WAF-1 ^expression in the clinical tumor samples.

There, tumors with positive pre-therapeutic p21^CIP/WAF-1 ^expression showed a better local tumor response. This is in line with the trend for longer survival in p21^CIP/WAF-1 ^expressing tumors in our previous study [6] and with data from ovarian cancer [19], rectal cancer [20,21], head and neck cancer [22], or pancreatic cancer [23]. As p21^CIP/WAF-1 ^is a direct transcriptional target of p53, it can be argued that therapy-naive tumors expressing p21^CIP/WAF-1 ^have an intact p53-regulatory pathway of cell cycle arrest, senescence and apoptosis. In fact, up-regulation of p21^CIP/WAF-1 ^may serve as a read-out for functional integrity of the p53 gene in malignant cells [2,24-26]. Nevertheless, a different picture is obtained when tumors are investigated not hours, but 4 to 6 weeks post-therapy: the persistence of highly p21^CIP/WAF-1^-expressing tumor cells 4 to 6 weeks after the completion of a neoadjuvant multimodal therapy is an adverse effect for survival [6]. It is therefore likely that this persistent increase of the amount of p21^CIP/WAF-1 ^expressing tumor cells is related to a selection process of resistant tumor cells by the neoadjuvant therapy that results in a stable increase of the p21^CIP/WAF-1 ^protein. Notably, we recently observed that cell cycle arrest via upregulation of p21^CIP/WAF-1 ^impedes IR-induced activation of a caspase-3 dependent apoptotic program [27]. This behaviour is mimicked in the HCT116 p21^CIP/WAF-1 ^k.o. model. There, p21^CIP/WAF-1 ^mediates resistance to radiochemotherapy but not heat-shock induced cell death.

Similarly to p21^CIP/WAF-1^, the differential effect of the pro-apoptotic Bax on sensitivity to radiochemotherapy and heat shock provides an additional molecular basis for combination of various treatment modalieties. Bax plays a key role in activating the mitochondrial apoptosis pathway upon various death stimuli. In cell line models, the presence of Bax was associated with increased apoptosis sensitivity to TRAIL, 5-FU [8], ceramide (von Haefen et al, 2002), the toxin calicheamicin [28] and IR [27] or the BH3-only protein Nbk/Bik [29], whereas other stimuli were Bax-independent [26,30]. In clinical samples, we found an intact p53/Bax-pathway to be associated with better survival [2-5]. Our present in vitro data therefore confirm that tumor cells without Bax are less sensitive to chemotherapy and IR. An even stronger impact of Bax loss was observed in heat shock induced apoptosis. Heat shock up to 43°C triggered apoptotic death in the Bax wild type HCT116 cells whereas Bax^-/- ^HCT116 cells were refractory to heat shock induced apoptosis. This is in line with a previous finding of our group showing that the enforced overexpression of Bak or Nbk/Bik sensitises for heat shock [15]. Most interestingly, the clinical data support these *in vitro *data strongly. Optimal hyperthermia was associated with a good tumor response only when tumors expressed high amounts of Bax whereas Bax-deficient tumors failed to respond to heat shock.

When compared to p21 and Bax, the p53 gene exhibited only a weak influence on sensitivity towards chemotherapy with 5-FU, irradiation and none towards heat shock. These *in vitro *data are in line with the clinical findings. Hence, up to now there is no convincing evidence that loss of p53 or mutational inactivation of p53 represents a negative prognostic factor or results in clinical resistance in carcinomas. This strengthens the assumption that p53 bypasses do exist and that essential p53 downstream effectors can be regulated by other means in case p53 fails.

## Conclusion

**Figure 6:**
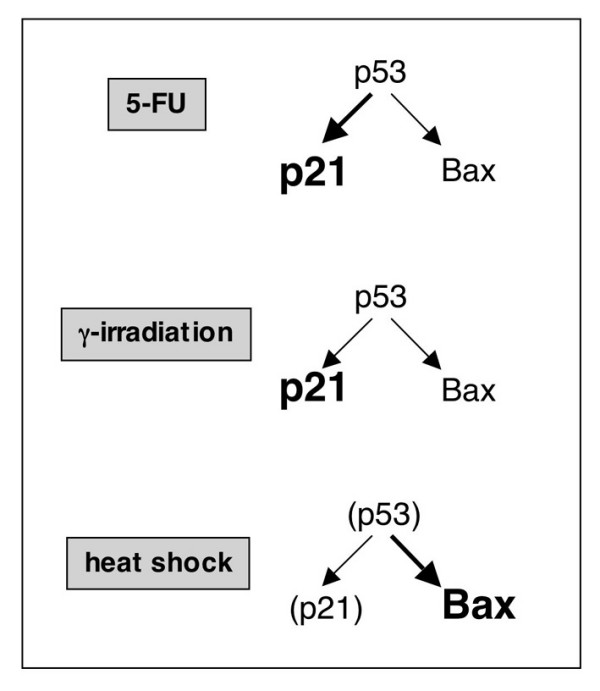
Model of individual genes (p53, p21^CIP/WAF-1 ^and Bax) showing differential impact on the cell death induction by 5-FU, IR or heat shock.

In conclusion, our data demonstrate that a systematic genetic and functional dissection of apoptosis and cell cycle regulatory pathways in tumor cells reveals differential cellular effects in various antitumor treatment modalities that have a direct impact on cancer therapy. A model of the differential sensitivities to 5-FU, γ-irradiation and heat shock with regard to Bax, p53 and p21^CIP/WAF-1 ^is outlined in figure 6. Notably, we demonstrated the relevance of our data in both clinical samples and in genetically defined, syngeneic HCT116 cells. These findings therefore provide an evidence-based cell biological model for the usefulness of combining heat shock/hyperthermia (which seems to be p21^CIP/WAF-1 ^and p53-independent, but Bax-dependent) with radiochemotherapy (which is p21^CIP/WAF-1^-dependent). Inclusion of additional pathway components and treatment modalities is, however, warranted towards the aim of a further individualisation of cancer therapy based on a molecular tumor profile.

## Competing interests

The author(s) declare that they have no competing interests.

## Authors' contributions

IS compiled the study, carried out the p53, p21, Bax analysis, performed the statistical analysis and drafted the manuscript. BR, PMS, PW, BH, HR treated the patients and collected the clinical data. SH reviewed the immunhistochemical analysis. BD participated in the design of the study, PD designed the study, performed data analyses and drafted the manuscript. All authors read and approved the final manuscript.

## Pre-publication history

The pre-publication history for this paper can be accessed here:



## Figures and Tables

**Table 1:** Patient characteristics and clinicopathological parameters in relation to response status.

			**HRCT**				**RCT**		
		n	Responder n = 18	Non-Res ponder n = 13	p	n	Responder n = 21	Non-Res ponder n = 14	p

**Age (year)**			61 ± 8*	58 ± 12*	n.s.		59 ± 7	57 ± 10	n.s.
**Gender**	male	23	16	7		18	11	7	
	female	8	2	6	n.s.	17	10	7	n.s.
**Infiltration depth**	uT3	26	13	13		32	19	13	
	uT4	5	5	0	n.s.	3	2	1	n.s.
**LN-involvement**	uN0	14	8	6		9	6	3	
	uNpos	17	10	7	n.s.	26	15	11	n.s.
**optimal HT**^1^		16	13	3					
**suboptimal HT**^2^		15	5	10	**0.01**				
**radiation dose**	< 45.94 Gy	2	1	1		1	0	1	
	≥ 45.94 Gy	29	17	12	n.s.	34	21	13	n.s.

HRCT: radiochemotherapy with heat shock/hyperthermia. RCT: radiochemotherapy without heat shock/hyperthermia. HT: local heat shock/hyperthermia. LN: lymph node

* mean ± SEM . n.s.: p > 0.05.

^1 ^optimal hyperthermia (temperature-time-interval above pre-defined value): cum min T_90 _≥ 40.5°C ≥ 120 min; ^2 ^suboptimal hyperthermia (temperature-time-interval below pre-defined value): cum min T_90 _≥ 40.5°C < 120 min
